# LMP1‐positive extracellular vesicles promote radioresistance in nasopharyngeal carcinoma cells through P38 MAPK signaling

**DOI:** 10.1002/cam4.2506

**Published:** 2019-08-22

**Authors:** Zhibao Zhang, Xuehui Yu, Zhuan Zhou, Bo Li, Jinwu Peng, Xia Wu, Xiangjian Luo, Lifang Yang

**Affiliations:** ^1^ Key Laboratory of Carcinogenesis and Cancer Invasion of Ministry of Education Departments of Oncology Xiangya Hospital, Central South University Changsha China; ^2^ Cancer Research Institute School of Basic Medicine Science Central South University Changsha China; ^3^ Pathology Department Xiangya Hospital, Central South University Changsha China

**Keywords:** extracellular vesicle, LMP1, nasopharyngeal carcinoma, P38, radioresistance

## Abstract

Radioresistance has been one of the impediments to effective nasopharyngeal carcinoma (NPC) therapy in clinical settings. Epstein‐Barr virus (EBV) encoded latent membrane protein 1 (LMP1) is expressed in NPC and has potent effects on radioresistance. It has been detected in extracellular vesicles (EVs) or exosomes and shown to promote tumor proliferation and invasive potential. However, whether LMP1‐positive EVs can confer radioresistance to cancer cells and the mechanism used to promote radioresistance need to be elucidated. In this study, the data showed that EVs derived from LMP1‐positive NPC cells could induce recipient NPC cell proliferation and invasion and suppress apoptosis, especially promoting radioresistance. In addition, LMP1 could increase the secretion of LMP1‐positive EVs. Furthermore, transmitted LMP1 subsequently performed its oncogenic functions through activating P38 MAPK signaling in recipient cells, and inhibiting P38 activity could efficaciously restore the sensitivity of NPC cells to ionizing radiation (IR). Finally, we found that LMP1‐positive EVs could promote tumor growth and P38 inhibition eliminates this promoting effect in vivo, and EV formation is associated with a poor prognosis in NPC patients. These results showed that a few cells expressing LMP1 could enhance the radioresistance of NPC cells through potentially impacting the infected host and also modulating the tumor microenvironment.

## Graphical abstract

In present study, we mainly demonstrated a new mechanism underlying NPC radioresistance that mediated by EBV‐LMP1‐positive EVs through P38 MAPK signaling. And the results showed that a few number of cells expressing LMP1 could enhance the radioresistance of NPC cells through both potentially impacting the infected host and also modulating the tumor microenvironment through the EVs.

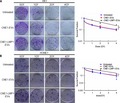

## 1 INTRODUCTION

Nasopharyngeal carcinoma (NPC), an Epstein‐Barr virus (EBV)‐associated malignancy that arises from the nasopharynx epithelium, has unique characteristics that make it highly distinct from other head and neck tumors. Compared to other cancer types, NPC is not common but usually happens in South China and Southeast Asia.^1^, ^2^ Radiotherapy always serves as a primary treatment for NPC. In recent years, innovations in radiation techniques have greatly improved disease control and the survival of early‐stage NPC patients. However, advanced NPC patients always show refractory radioresistance and approximately 34%‐52% of 5‐year survival rates.^3^, ^4^ Therefore, it is highly urgent to elucidate the underlying mechanisms of NPC radioresistance.

EBV, known as an oncogenic virus, participates in the pathogenesis of various human malignancies including NPC.^5^ EBV encoded latent membrane protein 1(LMP1) is a primary oncoprotein and plays pivotal roles in initiation and progression of NPC.^6^, ^7^ The activation of several intracellular signaling pathways by LMP1, such as the PI3K/Akt, JNK, MAPK/ERK, NF‐κB, and JAK/STAT etc, leads to the upregulation of multiple genes which are involved in modulation of cell proliferation, apoptosis, migration, and invasion.^8^ Importantly, our previous studies showed that suppressing LMP1 expression could enhance the radiosensitivity of NPC both in vivo and in vitro,^9^, ^10^ which demonstrated the importance of LMP1 in regulating the radioresistance of NPC.

Recently, intercellular communication mediated by extracellular vesicles (EVs) has been reported to be a new mechanism through which cancer cells can manipulate their microenvironment.^11^, ^12^ Based on the size and mode of release, EVs, as nanosized membrane vesicles, are classified into apoptotic bodies (>1 mm), microvesicles (MVs) secreted from the plasma membrane (>100 nm), and exosomes (about 100 nm) originated from multivesicular endosomes.^12^, ^13^ Exosomes and other EVs can be secreted by multiple cell types and transfer biological molecules (proteins, mRNAs, miRNAs) to other cells to modulate cell proliferation, angiogenesis, and tumor invasion.^14^, ^15^ However, the mechanism in biogenesis, secretion, and uptake of cancer EVs as well as the physiological significance of EVs composition are not yet understood.

Interestingly, LMP1’s localization to internal Golgi apparatus and MVB compartments and its packaging into exosomes for secretion have been investigated.^16^ Exosomes harboring LMP1 isolated from EBV‐infected B cells could be internalized by adjacent B lymphocytes, enhance proliferation, and drive B cell differentiation.^17^ LMP1‐positive exosomes enhance the motility and potential invasive ability of surrounding NPC tumor cells.^18^ Thus, it is likely that the LMP1 packaged into EVs or exosomes involves in oncogenesis by its multiple functions. However, whether EVs from LMP1‐positive NPC cells can confer radioresistance to sensitive cells and the mechanism involved in this process need to be elucidated.

In present study, we demonstrated the impacts of EVs from NPC cells expressing LMP1 on LMP1‐negative recipient cancer cells and verified that LMP1 could increase the secretion of LMP1‐positive EVs. Moreover, we found that P38 MAPK signaling was activated in recipient cells by EVs transmitting LMP1. We propose that LMP1‐positive EVs promote the radioresistance of NPC and that P38 MAPK participates in this process. These results showed that a few of cells expressing LMP1 could enhance the radioresistance of NPC cells through potentially impacting the infected host and also modulating the tumor microenvironment.

## 2 MATERIALS AND METHODS

### 2.1 Cell culture and reagents

CNE1‐LMP1 is a stable LMP1‐integrated cell line that was constructed from the LMP1‐negative NPC cell line CNE1.^19^ The LMP1‐negative cell lines, HK1 and HONE1, were established from squamous carcinomas of the nasopharynx.^20^, ^21^ RPMI 1640 medium (Gibco BRL) supplemented with 10% fetal bovine serum (FBS; HyClone) was used to culture the cells. Exo‐free FBS was purchased from SBI. The indicated antibodies were purchased: anti‐β‐actin (lot#A5441, Sigma‐Aldrich); anti‐p‐P38 (Thr180/Try182) and anti‐p‐JAK3 (lot#17852 and lot#16567, Santa Cruz Biotechnology); anti‐Calnexin, anti‐HSP70, anti‐p‐p65 NFκ8 (Ser536), anti‐SAPK/JNK, anti‐p‐ERK(1/2), and anti‐p‐Akt (ser473) (lot# 2433s, lot#3031s, lot#9252, and lot# 4370, Cell Signaling Technology); anti‐LMP1 (lot#M0897, DAKO); and anti‐CD63 (lot# ab134045 Abcam).

### 2.2 EV isolation

Cells were cultured to 70%‐80% confluence, washed twice with phosphate buffer saline (PBS), and cultured in RPMI 1640 medium supplemented with Exo‐free FBS for 48 hours. The cultured medium was then collected and precipitated twice at 3000 g for 20 minutes at 4°C to remove debris. Then, the supernatant medium was centrifuged in Amicon® Ultra‐15(3KD) filter units at 5000 g for 60 minutes. The supernatant medium was treated according to the steps described (EXOTC10A‐1, SBI).

### 2.3 TEM analysis of EV

EV‐containing pellets were resuspended in 1 × PBS, and 10 μL of suspension was added to 200 mesh formvar/carbon‐coated copper grids for 1 minute. Whatman paper was used to remove any residual fluid, and 10 μL of 2% uranyl acetate was added for negative staining. One minute later, staining was stopped by removing the fluids, and then the sample was dried for a few minutes. The results were observed with a transmission electron microscope (TEM) (FEI).

### 2.4 EV size distribution measurement

The particle size distributions of the EV samples were detected using a Zetasizer NanoS (Malvern Instruments).

### 2.5 Florescence microscopy to image EVs uptake by NPC cells

EVs extracted from CNE1‐LMP1 cells were prelabeled according to the instructions of a PKH67 cell line kit (lot#SLB5242V, Sigma‐Aldrich). PBS was used to rinse the EVs three times by condensing the volume with Amicon® Ultra‐15(3KD) filter units. HK1 cells were seeded on a Millicell EZ slide (Millipore) (~1000/well, 500 μL) and cultured for 8 hours to allow the cells to adhere to the cubicle. The HK1 cells were washed three times with PBS and cultured with 25 μg of PKH67‐labeled CNE1‐LMP1‐derived EVs for 24 hours, and the results were imaged using a confocal microscope (LSM 510 META, Carl Zeiss).

### 2.6 Colony formation assay

A gradient number of HK1 or HONE1 cells were seeded into 6‐well dishes and cocultured with different EVs or SB203580. After the cells were attached to the plates, the cells were irradiated with X‐ray (1 Gy, 2 Gy, or 4 Gy), then followed by further culturing for 2‐3 weeks to allow colony formation. Colonies (>50 cells) in dishes were stained with 0.0125% crystal violet (Sigma‐Aldrich). Plating efficiency was analyzed as described.^22^

### 2.7 Viability assay

Cell viability was measured using an MTS kit (lot#G971A, Promega). Cells were cultured in a 96‐well plate, and the assay solution was added for an incubation of 1.5 hours in the dark. Amicroplate reader (BioTek ELx800) was used to measure the results at 490 nm.

### 2.8 Apoptosis analysis by flow cytometry

Cells were seeded into 6‐well plates and cocultured with different EVs. Then, cells (1‐5 × 10^5^) were collected and resuspended with binding buffer (500 μL). Subsequently, Annexin V‐PE (1 μL) and 7‐AAD (5 μL) were added into cell suspensions, and mixed for 5‐15 minutes. Finally, the cells were subjected to flow cytometry (FCM, MoFlo XDP, Beckman) analysis within an hour of staining.

### 2.9 Cell invasion assay

Cell invasion assay was conducted using Matrigel 24‐well invasion chambers, with 8.0‐μm pore filters coated with Matrigel on the upper surface (BD Biosciences). In brief, 1 × 10^5^ cells with serum‐free medium were seeded in the upper chamber, and 10% FBS‐containing medium was added to the lower chamber. After 24 hours of incubation, the cells were visualized by staining with crystal violet solution. The cells and Matrigel on the top surface of the filter were removed and the invasive cells attached to the bottom surface of the filter were measured by light microscope. The data are presented as the average number of cells in randomly chosen fields.

### 2.10 Quantitative RT‐PCR

Total RNA was obtained and performed reverse transcription as described before.^19^ Quantitative PCR was performed with iTaq^TM^ SYBR Green Supermix with ROX (172‐5850, Bio‐Rad) using an ABI 7500 instrument. The primers used to detect LMP1 were 5′‐CGTTATGAGTGACTGGACTGGA‐3′ (forward) and 5′‐TGAACAGCACAATTCCAAGG‐3′ (reverse). The primers for detecting β‐actin were 5′‐CATGTACGTTGCTATCCAGGC‐3′ (forward) and 5′‐CTCCTTAATGTCACGCACGAT‐3′ (reverse).

### 2.11 Western blotting analysis

Proteins were extracted from IP lysis buffer‐treated cells (Pierce™, Thermo Fisher) with cocktail and PhosStop (Roche). Total cell lysates in sodiumdodecyl sulfate (SDS) (Sigma‐Aldrich) loading buffer were denatured, and separated by SDS‐polyacrylamide gel electrophoresis (SDS‐PAGE), the sample were transferred to PVDF membranes (General Electric). The membranes were blocked with 5% milk in Tris‐buffered saline‐Tween 20 (TBST) and incubated overnight at 4°C with primary antibodies. The membranes were then washed and incubated with secondary antibodies for 1 hour at room temperature. The membranes were washed three times with TBST again and visualized with the chemiluminescence detection kit (Pierce ECL, Thermo Fisher Scientific).

### 2.12 Animal experiments

Animal experiments were approved by the Animal Care Committee of Xiangya Hospital in accordance with Institutional Animal Care and Use Committee guidelines. HONE1 cells (1 × 10^7^) that mixed with 100‐μg EVs isolated from the culture media of CNE1 cells or CNE1‐LMP1 cells, respectively, were injected into 6‐week‐old female athymic nude mouse (BALB/C) to establish xenografts, and then 100‐μg EVs was injected every 2 days in the vicinity of the subcutaneous tumors. When tumor volume reached about 60 mm^3^, animals were intraperitoneally injected with SB203580 (5 mg kg^−1^ d^−1^) for about 15 continuous days. Tumor formation was examined every 3 days. At the indicated time points, animals were sacrificed, and tissues were collected and fixed with 10% buffered formalin for immunohistochemistry (IHC) analysis.

### 2.13 Immunohistochemistry (IHC)

NPC tissue chip was purchased from Pantomics, and paraffin‐embedded tumor tissue samples with clinical details and follow‐up data of NPC patients (from 2012 to 2017) were collected from the Pathology Department of Xiangya Hospital. IHC was performed using a Histomouse SP Broad Spectrum DAB kit (Invitrogen‐Zymed). The operation steps of IHC are strictly in accordance with previous reports.^19^ All sections were immunostained independently and reviewed by two pathologists (BL and JP).

### 2.14 Statistical analysis

Data analysis used SPSS software (version 21.0; SPSS). The *t *test of independent samples was used to compare the quantity data one by one. The repeated measurement analysis of variance was analyzed with one‐way ANOVA. The correlation between the two samples was compared using Spearman correlation coefficient. The Kaplan‐Meier method was used to estimate overall survival. Values are expressed as the mean ± SE of three individual experiments. A value of *P* < .05 was considered statistically significant.

## 3 RESULTS

### 3.1 NPC cell‐derived EVs are internalized by recipient cancer cells

**Figure 1:**
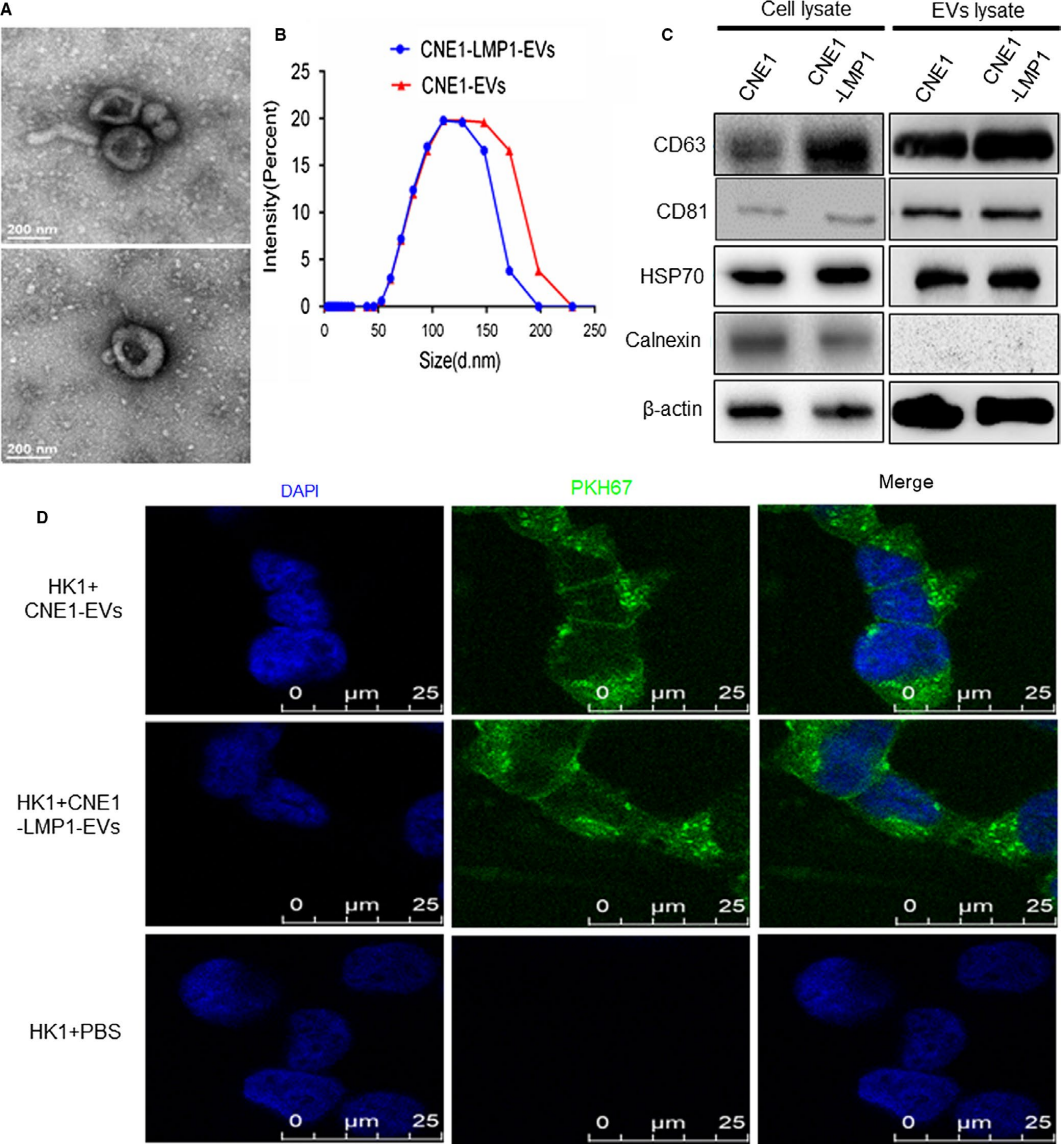
Nasopharyngeal carcinoma (NPC) cell‐derived extracellular vesicles (EVs) are internalized by recipient cancer cells. A, Representative TEM images of the EVs secreted by CNE1‐LMP1 cells. B, Size distribution analysis of the EVs isolated from the culture media of CNE1 and CNE1‐LMP1 cells. C, Western blot analysis of the indicated proteins as putative negative (calnexin) or positive (CD63, CD81, and HSP70) markers of EVs. D, Immunofluorescence analysis of PHK67‐labeled CNE1 and CNE1‐LMP1 cell‐derived EVs taken up by HK1 cells. Scale bar, 25 μm

EVs were isolated from the supernatants of CNE1 and CNE1‐LMP1 cells using Exo‐Quick‐TC and analyzed with TEM, and the size distribution was confirmed using nanosight analysis. We noted the presence of 100‐150 nm vesicles (Figure 1A), the size distribution ranged from 50 to 200 nm, and the peak was approximately 100‐150 nm (Figure 1B), which was consistent with the peak in the preliminary findings.^23^ Then, we characterized the EV's protein lysates by Western blot analysis for CD63, CD81, and HSP70 (putative markers for EVs) as well as calnexin (marker for the endoplasmic reticulum). As shown in Figure1C, the data also confirmed that the isolated vesicles were mostly EVs. To examine whether NPC cell‐derived EVs are taken up by cancer cells, we stained the EVs with PKH67 dye and incubated them with HK1 cells for 24 hours. The results (Figure 1D) showed that green fluorescence spots were found in the HK1 cells, but no fluorescence signal in control group without EVs, which indicated that NPC cells are able to internalize EVs.

### 3.2 CNE1‐LMP1 cell‐derived EVs promote radioresistance, proliferation, and invasion and suppress apoptosis in recipient cancer cells

**Figure 2:**
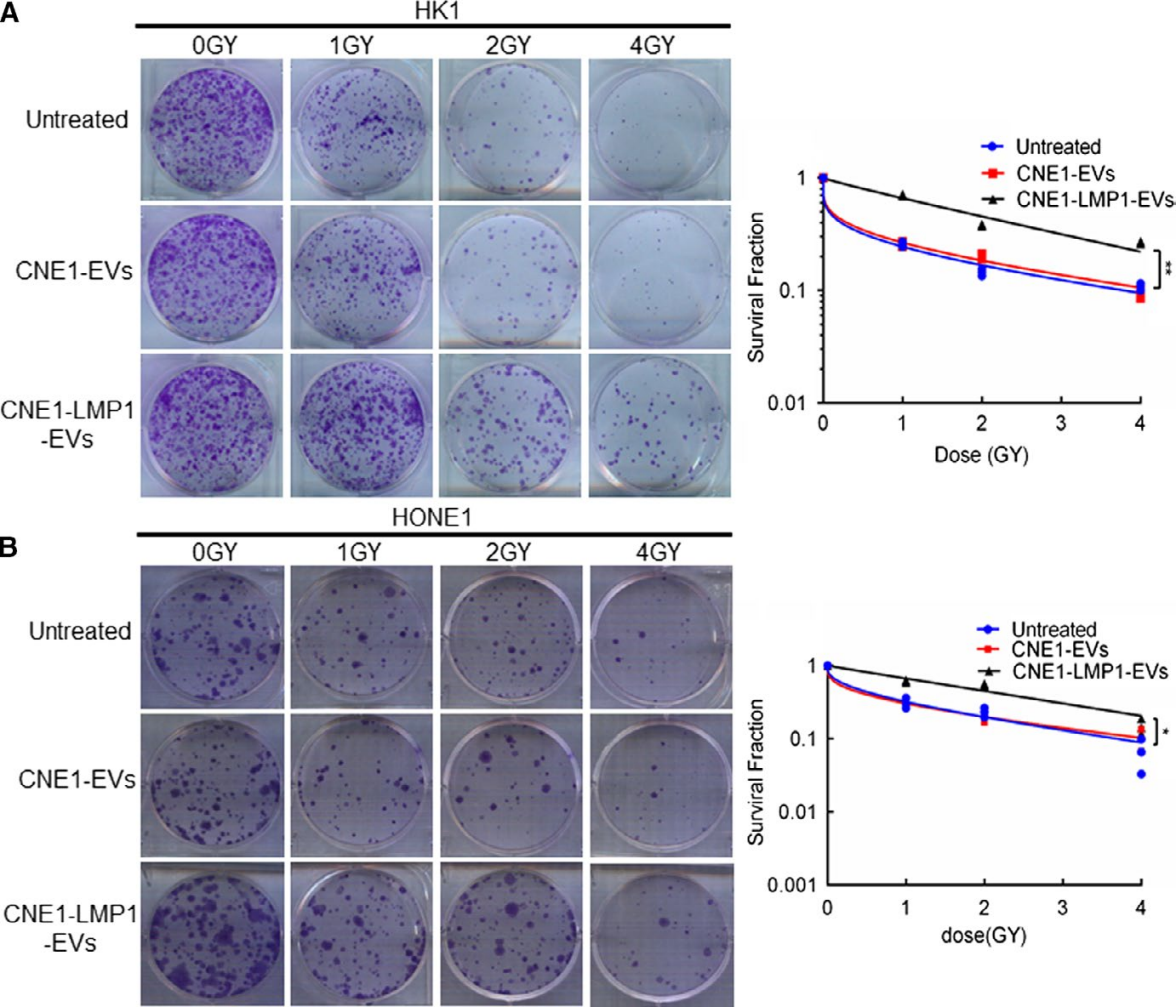
CNE1‐LMP1 cell‐derived extracellular vesicles (EVs) promote radioresistance in recipient cancer cells. HK1 and HONE1 cells were treated with IR at 0, 1, 2, or 4 Gy and cultured with EVs from CNE1 or CNE1‐LMP1 cells for 2 weeks. Then, fixation, staining, and colony quantification were performed. A and B, Left: representative images of hexamethylpararosaniline‐stained colonies of HK1 or HONE1 cells. Right: survival curves of HK1 or HONE1 cells fit to the data using a multi‐target single‐hit survival model of radiosensitivity. Data are expressed as the mean ± SD of three experiments. *P < .05, **P < .01

To illustrate the effects of EVs from NPC cells expressing LMP1 on recipient cancer cells, we first investigated the influence of EVs on cancer cell radioresistance. HK1 and HONE1 cells were incubated with 50 μg/mL EVs from the culture medium of CNE1 or CNE1‐LMP1 cells, and a colony formation assay was performed. The results are shown in Figure 2A and B. The ability of colony formation after ionizing radiation (IR) was essentially unchanged between the groups treated with the EVs from CNE1 cells and the untreated groups but was significantly enhanced in the groups treated with the EVs from CNE1‐LMP1 cells (*P* < .01 for HK1 and *P* < .05 for HONE1), which illustrated the functions of EVs derived from LMP1‐positive NPC cells in mediating the radioresistance of NPC cells.

**Figure 3:**
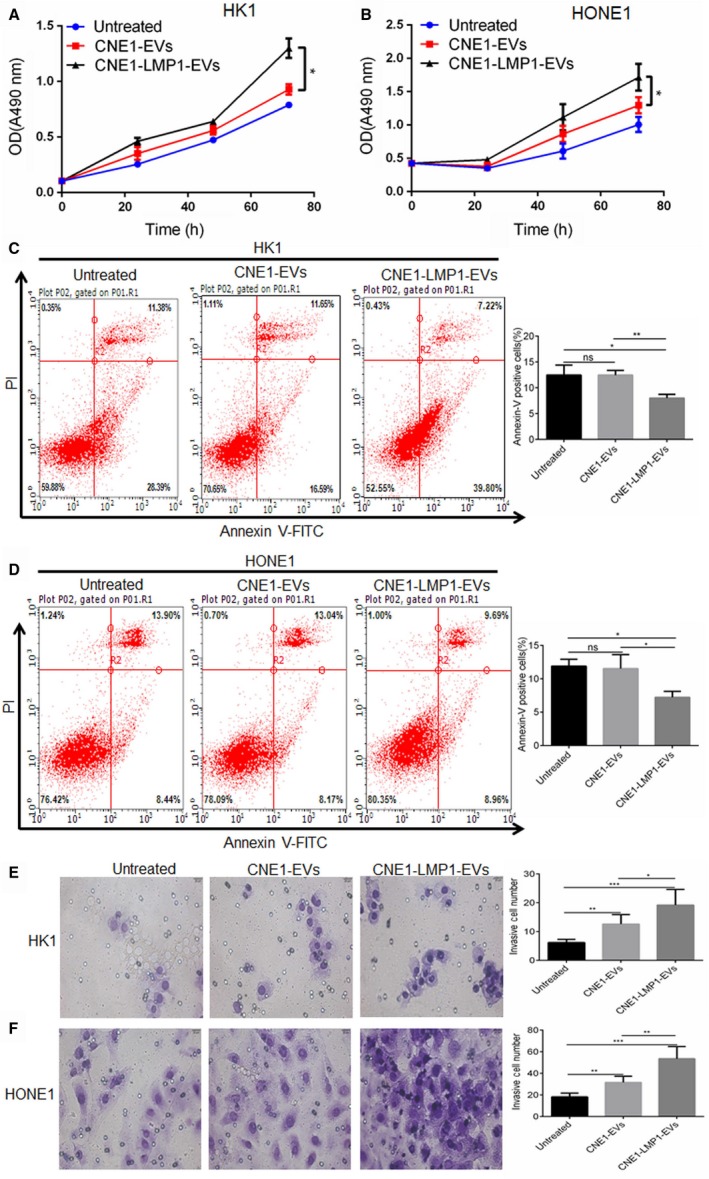
CNE1‐LMP1 cell‐derived extracellular vesicles (EVs) enhance the proliferation and invasive potential and reduce the apoptosis rate of recipient nasopharyngeal carcinoma (NPC) cells. HK1 and HONE1 cells were treated with 50 μg/mL EVs from CNE1 or CNE1‐LMP1 cells for 24 h. A and B, An MTS assay was used to analyze the cell viability of HK1 or HONE1 cells after treatment with the EVs. C and D, Left: representative graphs showing the proportion of HK1 or HONE1 cells that were positively stained with Annexin‐V. Right: analysis of three independent experiments assessing the proportion of Annexin‐V‐positive cells. E and F, Left: representative graphs showing the invasion of HK1 or HONE1 cells through the 0.4‐μm pore of the transwell. Right: statistical analysis of three independent experiments assessing the number of HK1 or HONE1 cells in five visual fields. The results are presented as the mean ± SD.*P < .05, **P < .01, ***P < .001, ns not significant

Furthermore, we examined the influence of different cell‐derived EVs on proliferation, apoptosis, and invasion of recipient cancer cells. HK1 and HONE1 cells were incubated with 50 μg/mL EVs from the culture medium of CNE1 or CNE1‐LMP1 cells, and a cell viability assay was performed. The data showed that the HK1 and HONE1 cells treated with the EVs derived from CNE1 cells demonstrated an increase in cell proliferation compared to the untreated cells; however, this increase was less dramatic than that of the cells treated with the EVs derived from CNE1‐LMP1 cells (Figure 3A,B). These results suggested that although EVs from both LMP1‐positive and LMP1‐negative NPC cells can promote recipient cancer cell proliferation, the differences in the contents of EVs result in variations in their functions.

Furthermore, we conducted an apoptosis assay with the HK1 and HONE1 cells treated with the CNE1‐ or CNE1‐LMP1 cell‐derived EVs for 24 hours. The results showed that the proportions of apoptotic cells for the HK1 and HONE1 cells treated with the EVs from CNE1‐LMP1 cells were much lower than those for the cells treated with the EVs from CNE1 cells or those for the untreated cells (Figure 3C,D).These data demonstrated that EVs secreted from LMP1‐positive NPC cells can promote antiapoptotic processes in recipient cancer cells.

In addition, to determine whether NPC‐derived EVs promote the invasive potential of cancer cells, HK1 and HONE1 cells were preincubated with EVs isolated from different cells, and seeded in the top chamber of a transwell insert, then allowed to migrate for another 24 hours. As shown in Figure 3E and F, NPC cells treated with different EVs exhibited significantly more invasiveness than the untreated cells. We also observed that the numbers of invasive HK1 and HONE1 cells treated with the CNE1‐LMP1 cell‐derived EVs were greater than those of the cells treated with the CNE1 cell‐derived EVs (*P* = .0490 and 0.0044, respectively). These results suggested that EVs secreted by NPC cells, especially LMP1‐positive cells, could confer enhanced invasion potential to recipient cells.

### 3.3 LMP1 promotes the secretion of EVs containing LMP1

**Figure 4:**
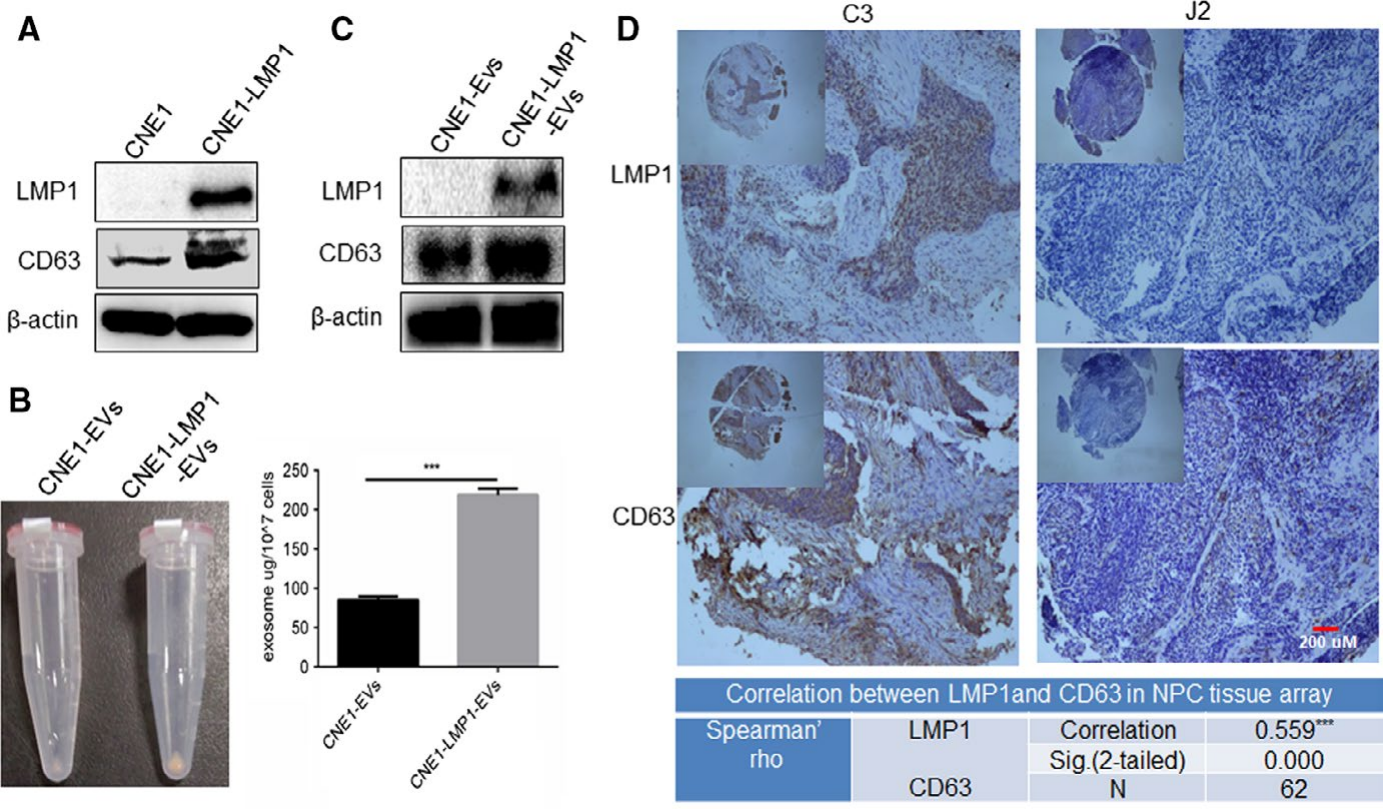
LMP1 promotes the secretion of extracellular vesicles (EVs) containing LMP1. A, Western blot analysis of CD63 and LMP1 expression in CNE1 and CNE1‐LMP1 cells. B, Right: representative image of precipitations of EVs derived from CNE1 or CNE1‐LMP1 cells. Left: statistical analysis of a BCA protein assay of the EVs. The results are presented as the mean ± SD. C, Western blot analysis of LMP1 expression in EVs derived from CNE1 and CNE1‐LMP1 cells. D, Upper: representative images of IHC staining for CD63 and LMP1 in a commercial NPC tissue array. Magnification: 40× or 200×. The tissue sample C3 exhibited high expression of LMP1 and CD63, whereas the tissue sample J2 exhibited low levels of LMP1 and CD63. Lower: statistical analysis of the correlation between LMP1 and CD63 using Spearman's Rho. ***Correlation is significant at the 0.001 level (two‐tailed)

To further elucidate the role of LMP1 in the EVs‐mediated radioresistance of NPC, we conducted various methods to test the influence of LMP1 on EV secretion and interrogated the identity of the EVs. First, a Western blot assay confirmed that the expression of the CD63 protein, a marker of EVs, was higher in CNE1‐LMP1 cells than in CNE1 cells (Figure 4A), which suggested that LMP1 can promote EV formation. Then, we cultured CNE1 and CNE1‐LMP1 cells with Exo‐depleted FBS for 24 hours and collected the culture media for EV extraction using the Exo‐Quick‐TC™ EV precipitation solution. As shown in Figure 4B, representative photos of the precipitated EV pellet showed that CNE1‐LMP1 cells (1 × 10^6^) secreted more EVs than the same number of CNE1 cells. EV secretion was observed to be increased by three‐ to fourfold in the CNE1‐LMP1 cells compared to the CNE1 cells by a protein assay (*P* < .001). Furthermore, we interrogated the identity of the EVs secreted following LMP1 expression. The Western blot assay showed that LMP1 accumulated partly in EVs, and LMP1 enhanced the production of CD63+ EVs (Figure 4C). Furthermore, an IHC assay was performed using a commercial NPC tissue array to detect protein expression. As shown in Figure 4D, CD63 expression was higher in LMP1‐positive tissue than LMP1‐negative tissue, and there was a significant positive correlation between the expression of the two proteins (correlation coefficient = .559, *P* < .001). These data demonstrated that LMP1‐positive NPC cells facilitated the secretion of EVs and that purified EVs contained LMP1.

### 3.4 P38 MAPK pathway activation is responsible for LMP1‐positive EV‐mediated NPC radioresistance

**Figure 5:**
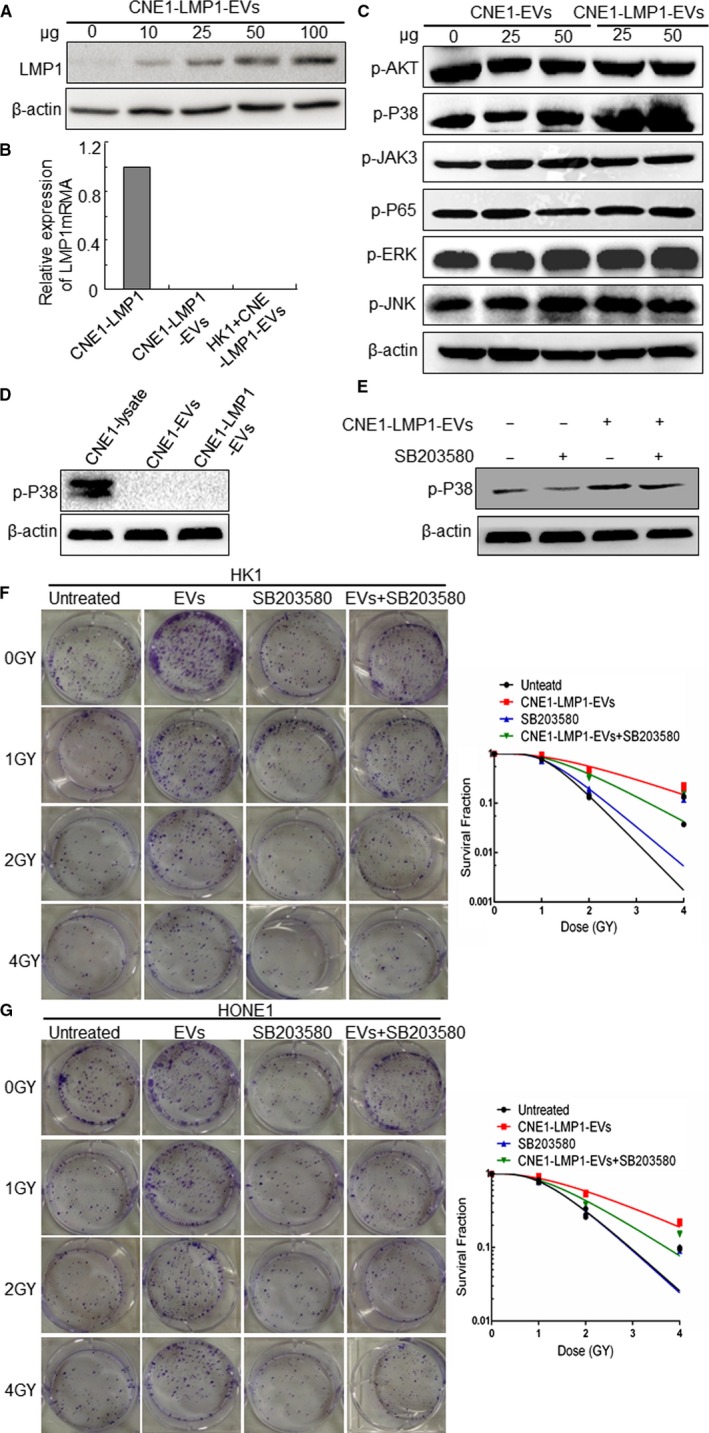
Extracellular vesicles (EVs) from LMP1‐positive nasopharyngeal carcinoma (NPC) cells transmit LMP1 to recipient cells and activate the P38 signaling pathway. A, Western blot analysis of LMP1 expression in recipient HK1 cells incubated with 0, 10, 25, 50, or 100 μg of EVs from CNE1‐LMP1 cells. B, Evaluation of the LMP1 mRNA levels in CNE1‐LMP1 cells, EVs derived from the CNE1‐LMP1 cells, and HK1 cells treated with the EVs by quantitative RT‐PCR. C, Western blot analysis of the indicated proteins activated by LMP1 in HK1 cells cultured with EVs from CNE1 or CNE1‐LMP1 cells. D, Assessment of the p‐P38 level in EVs derived from CNE1 and CNE1‐LMP1 cells. Total protein from CNE1‐LMP1 cell lysates was used as a positive control. E, The inhibitory effect of SB203580 (5 μmol/L) on P38 in HK1 cells. F, HK1 cells were treated with EVs (from CNE1‐LMP1 cells), SB203580, or SB203580 + EVs after irradiation at 0, 1, 2, or 4 Gy. The cells were incubated for 2 weeks before fixation, staining, and colony formation quantification. Left: representative pictures of the colony formation assay. Right: the survival fraction curve was fitted to the data. G, Left: representative HONE1 cell colony formation. Right: the survival fraction of the HONE1 cell colony

LMP1‐positive NPC cells could secrete EVs containing LMP1 and promote the radioresistance of recipient cancer cells. First, to gain insights into the mechanisms involved in this process, we tested whether EVs could transfer LMP1 to recipient cells. After HK1 cells were cocultured with increasing concentrations of CNE1‐LMP1 cell‐derived EVs for 24 hours, as shown in Figure 5A, a gradual increase in the LMP1 level was detected in the recipient HK1 cells, and the results suggested that the LMP1 protein level increased after EV internalization. To determine whether the LMP1 protein level increase was the result of horizontal transfer or EV‐induced overexpression, we detected the LMP1 mRNA levels in CNE1‐LMP1 cells, EVs derived from the CNE1‐LMP1 cells, and HK1 cells treated with the EVs. The results showed that LMP1 mRNA transcripts only existed in the CNE1‐LMP1 cells, indicating that the increased LMP1 level in the recipient cells occurred via horizontal transfer by EVs (Figure 5B).

LMP1 can activate multiple signaling pathways involved in proliferation, apoptosis, metastasis, and radioresistance, such as AKT, JAK3, P38 MAPK, JNK, ERK, and NF‐κB p65 signaling, in NPC tumor cells.^7^ To determine which pathway is most critical for the enhancement in radioresistance induced by LMP1‐positive EVs in recipient cancer cells, we detected the expression of activating signaling molecules in the recipient cells after LMP1‐positive EV treatment. The results showed that the expression of phosphorylation‐activating P38 increased obviously, whereas the levels of the other activating signaling proteins in the group treated with the CNE1‐LMP1 cell‐derived EVs changed very little compared with those of the untreated group or the group treated with the CNE1 cell‐derived EVs (Figure 5C). In addition, the results showed that there was no expression of activating p38 in the LMP1‐positive EVs (Figure 5D), which indicated that activating P38 is endogenous to the recipient cancer cells, not transferred by the EVs. These results suggested that LMP1‐positive EVs can stimulate P38 MAPK signaling through the exosomal transfer of LMP1 to recipient cells.

Furthermore, we investigated the roles of the EV‐mediated activation of P38 MAPK signaling in radioresistance and whether the inhibition of activating P38 can restore the sensitivity of NPC cells to IR. Western blot analysis was conducted to test the effect of the inhibitor SB203580 on activating P38 in HK1 cells, and the results indicated that SB203580 decreased the p‐P38 protein level effectively induced by the LMP1‐positive EVs (Figure 5E). Then, a colony assay revealed that the inhibition of P38 MAPK signaling in the recipient cells treated with SB203580 (5 μmol/L) significantly decreased the colony numbers of the HK1 and HONE1 cells treated with the CNE1‐LMP1 cell‐derived EVs (Figure 5F,G), which indicated that inhibiting P38 activity can restore the radiosensitivity of NPC cells transformed by LMP1‐positive EVs. These data further demonstrated that the activation of P38 MAPK signaling was responsible for the radioresistance induced by the EVs transmitting LMP1 to the recipient cancer cells.

### 3.5 P38 inhibitor eliminates the promotion of tumor growth by LMP1‐positive EVs in vivo

**Figure 6:**
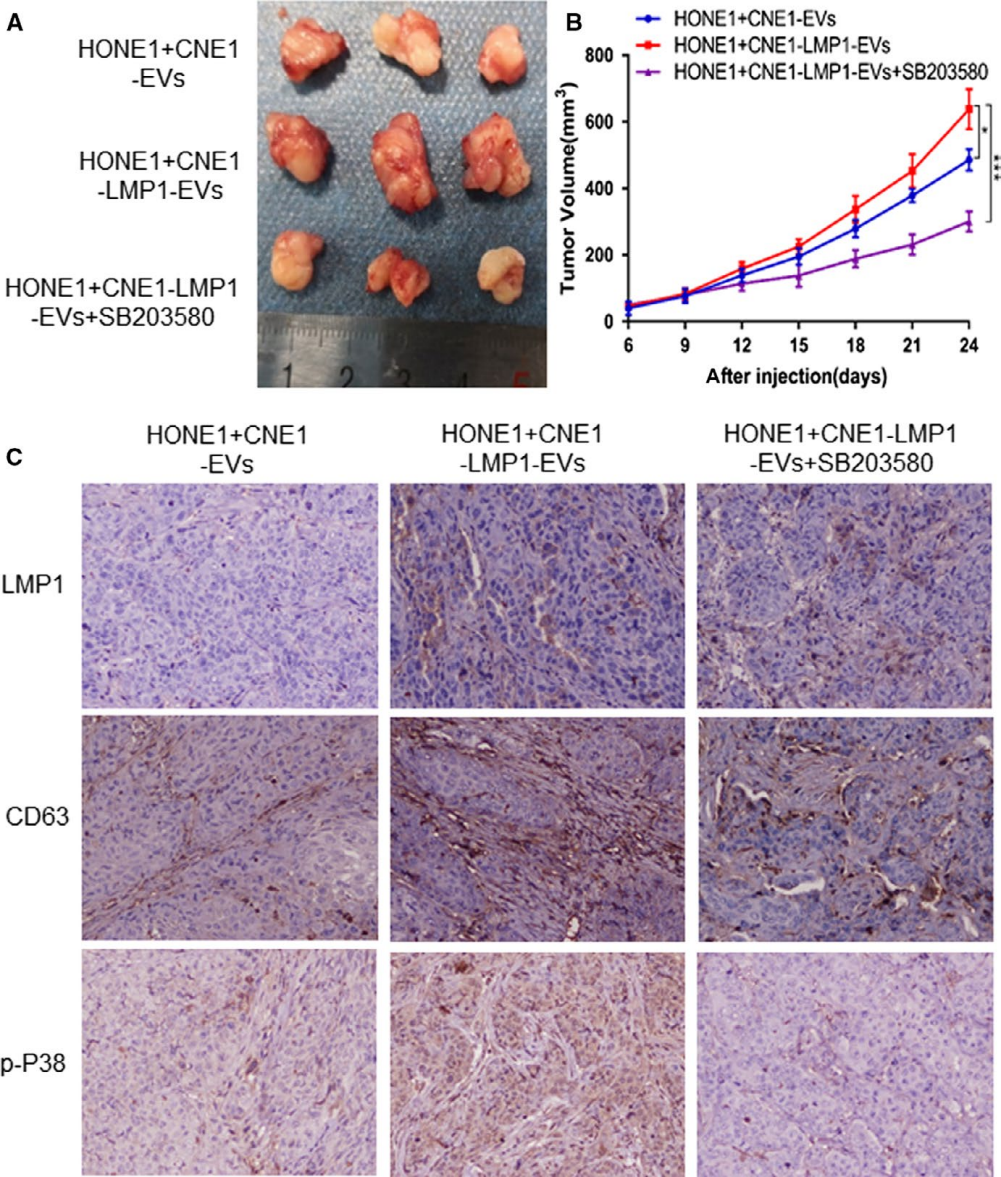
P38 inhibitor eliminates the promotion of tumor growth by LMP1‐positive extracellular vesicles (EVs) in vivo. Female BALB/c nude mice were subcutaneously inoculated with 1 × 10^7^ HONE1 cells. Xenografts treated with CNE1‐EVs, CNE1‐LMP1‐EVs, and CNE1‐LMP1‐EVs plus SB203580, respectively. A, At the end of the experiment, the mice were sacrificed and the tumors were separated. Tumor mass of each group was shown in the graph. B, Tumor volume was examined every 3 d and shown in the graph. C, Representative images of IHC staining assay for LMP1, CD63, and p‐P38. Magnification, 200×

For verifying that CNE1‐LMP1 cell‐derived EVs promote tumor growth in vivo, and whether the activation of P38 MAPK signaling was responsible for this promoting effect, the animal experiments were conducted. As shown in Figure 6A and B, the results indicated that LMP1‐positive EVs promote the tumor growth comparing with the EVs derived from CNE cells culture media. However, treatment with the p38 MAPK inhibitor SB203580 resulted in significant reduced tumor volume (*P* < .001). IHC staining showed that after the treatment of LMP1‐positive exosomes, the expression of LMP1 was obvious in the tumor tissues, and the expression of CD63 which represents the EVs secretion in tumor tissues and stroma was increased as well. The results also showed that p‐P38 was successfully inhibited in SB203580‐treated group (Figure 6C). These data further demonstrated that P38 inhibitor eliminates the promotion of tumor growth by LMP1‐positive EVs in vivo.

### 3.6 LMP1 induces EV formation and correlates with a poor prognosis in NPC patients

**Figure 7:**
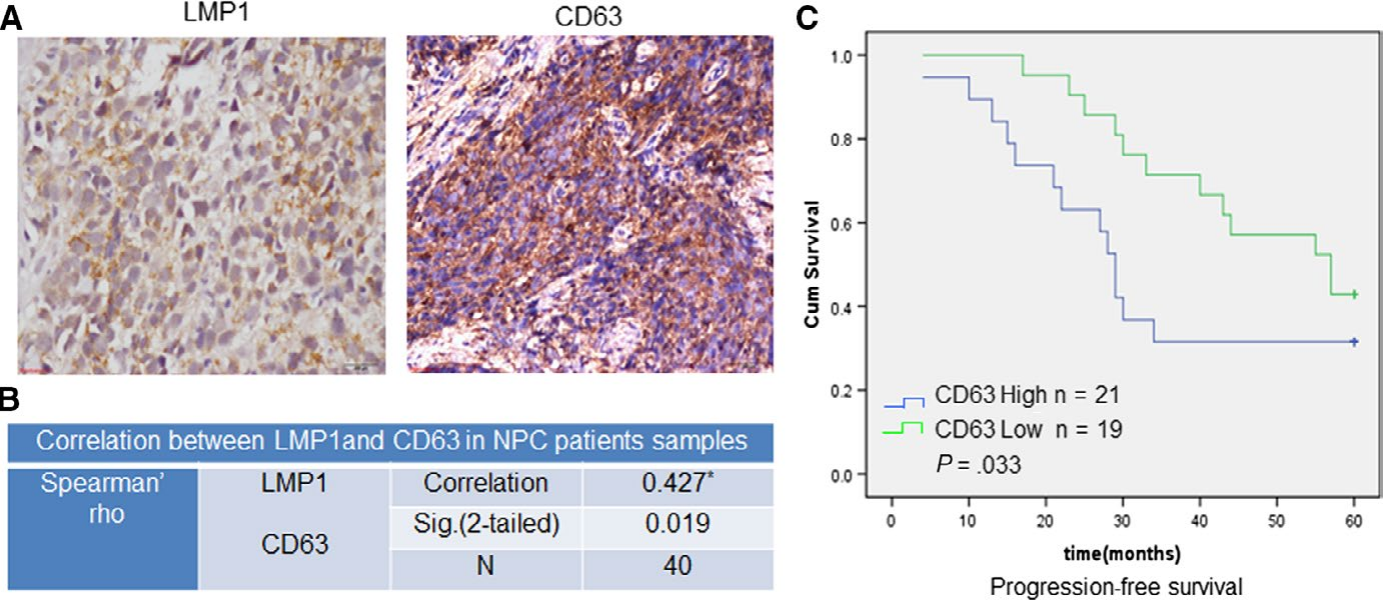
LMP1 induces EV formation and correlates with a poor prognosis in NPC patients. A, Representative image of the IHC staining for LMP1 and CD63 in NPC tissue samples from patients from Xiangya Hospital. B, Analysis of the correlation between CD63 and LMP1 expression in NPC tissue. C, The progression‐free survival times of NPC patients with high or low expression levels of CD63 were estimated with the Kaplan‐Meier method by log‐rank test. *Correlation is significant at the 0.05 level (two‐tailed)

Based on previous observations of the vital role of LMP1‐positive EVs in NPC radioresistance in vitro, we next analyzed the possible correlation between LMP1 and EV secretion in 40 clinical patient samples. The results of an IHC analysis showed a significant positive correlation between LMP1 and CD63 expression (Figure 7A,B). These data implied that communication via EVs was enhanced in the LMP1‐positive NPC tumor tissue samples, which is consistent with the result of the commercial NPC tissue array shown in Figure 4D.

To further explore whether this enhanced communication between NPC cells via EVs promotes NPC radioresistance, a retrospective analysis of the correlation between CD63 and progression‐free survival (PFS) in NPC patients receiving IR therapy was performed. As shown in Figure 7C, compared with CD63‐positive group, CD63‐negative group showed a longer median survival time (*P* = .033). These results demonstrated a clear correlation between the enhanced communication of NPC cells mediated by EVs and the poor prognosis of NPC patients receiving IR therapy.

## 4 DISCUSSION

A large amount of evidence has certified the roles of EVs in the modulation of the tumor microenvironment, including altering the immune response and strengthening tumor progression.^24^, ^25^ Previous studies have shown that the viral oncogene LMP1 can be transmitted between cells by EVs or exosomes, and this transmission has relations with EBV‐induced oncogenesis.^26^ Aga et al^18^ reported that HIF1α‐carried exosomes were secreted from LMP1‐positive NPC cells and its uptake by surrounding tumor cells could promote cancer cell invasion. Meckes et al found that through the intercellular transfer of LMP1, signaling molecules, and viral miRNAs, exosomes may manipulate the tumor microenvironment and accordingly influence the growth of neighboring cells.^27^ However, the present study is the first to reveal that the EVs from LMP1‐positive NPC cells not only enhanced proliferation and invasiveness while inhibiting apoptosis but also promoted radioresistance in recipient NPC cells. Furthermore, clinical studies recently have displayed an increasing amount of circulating EVs in patients with late‐stage cancer, which were corresponded with disease progression to a certain extent.^28^, ^29^, ^30^ Here, we demonstrated that the expression of CD63, which is a marker of EV formation, was associated with a poor prognosis in NPC patients.

A series of studies by our group recently confirmed that LMP1, the major oncogenic protein encoded by EBV,^7^, ^31^ contributes to the NPC radioresistance through promoting radioresistance. LMP1 might affect tumor angiogenesis via JNKs/HIF‐1 pathway, and regulate glycolysis through upregulation of a rate‐limiting enzyme hexokinase 2 (HK2), or inhibit telomerase activity of NPC cells.^19^, ^20^, ^32^ In addition, we proved that the reactivation of AMPK by metformin in LMP1‐positive NPC cells could substantially reverse radioresistance both in vitro and in vivo, implicating that AMPK is responsible for the LMP1‐mediated radioresistance of malignancies.^9^ LMP1‐induced cancer stem cell (CSC)‐like properties in NPC cells were also shown to contribute to radioresistance by our studies.^33^ However, these studies mostly focused on cell‐autonomous endocellular regulation by LMP1. As LMP1 expression cannot be detected in all tumor cells or tissues, by paracrine action, a part of LMP1‐positive NPC cells could alter and impact the growth control of other negative cells within a tumor. Our present study demonstrated a new mechanism underlying NPC radioresistance mediated by LMP1‐positive EVs.

Previous studies have shown the increasing variety of biologically active molecules that can be transported intercellularly by EVs.^34^ In fact, many signaling molecules can be transferred to recipient cells by LMP1‐positive EVs as well. For example, LMP1 could improve the concentration of EVs with FGF‐2, a potent angiogenic factor, as well as the release of this molecule through EVs.^35^, ^36^ Meckes et al^27^ also found that LMP1 expression enhances the secretion of EGFR in exosomes. Another study demonstrated that LMP1‐positive exosomes contain high levels of HIF1α, therefore support potential tumor migration and invasion.^18^ These findings suggest that the content and properties of EVs could be modulated by effects of LMP1 on cellular expression. Moreover, both intra‐ and intercellular signaling capabilities were linked through exosomal trafficking of LMP1. For instance, the blocking of exosomal LMP1 secretion caused downstream intracellular NF‐κB overstimulation within cells.^37^ Besides, the transfer of LMP1‐containing EVs in naive recipient cells leads to the activation of PI3K/Akt and MAPK/ERK signaling pathways.^27^These experiments suggest that EVs containing LMP1 have an important impact on intercellular communication under the environment of viral infection and may promote the carcinogenic effect of EBV. However, the present study demonstrated that the activation of P38 MAPK signaling was responsible for the radioresistance and tumor growth induced by the EVs transmitting LMP1 to recipient NPC cells, which is meaningful for improving the understanding of the function and mechanisms of the exosomal trafficking of LMP1.

In present study, the results directly displayed that LMP1‐positive NPC cells produced more EVs than LMP1‐negative NPC cells, and implicated clinical significance of the exosomal packaging of this viral oncoprotein. However, the mechanisms of LMP1 trafficking into EVs have not been clarified yet. Several studies have proposed that CD63 may account for the mechanism of LMP1 incorporation into EVs and that caveolin is responsible for EV secretion.^37^, ^38^, ^39^, ^40^ We still believe that more investigations are needed to further uncover the mechanisms of LMP1 trafficking into EVs. Moreover, new insights into the roles and functions of LMP1‐positive EVs in recipient cells are also needed in the near future.

In conclusion, this study confirmed that EVs derived from LMP1‐positive NPC cells could confer radioresistance to recipient NPC cells by activating P38 MAPK signaling, which suggested that a small proportion of cells expressing LMP1 could enhance the radioresistance of NPC cells through potentially impacting the infected host and also modulating the tumor microenvironment.

## CONFLICT OF INTEREST

The authors declare no potential conflict of interest.

## Data Availability

I confirm that my article contains a Data Availability Statement even if no data is available (list of sample statements) unless my article type does not require one.
